# High-intensity interval training and strength conditioning in patients with chronic lymphocytic leukemia: a systematic review

**DOI:** 10.1186/s13643-025-02764-9

**Published:** 2025-05-23

**Authors:** Pedro M P Cunha, Ricardo J Ribeiro, Andreia Pizarro, Jorge Mota, Jose C D Ribeiro

**Affiliations:** 1 Faculty of Sports, FADEUP) and Laboratory for Integrative, Research Center in Physical Activity, Health and Leisure (CIAFEL)University of PortoTranslational Research in Population Health (ITR), Porto, Portugal; 2 i3S-Instituto de Investigação E Inovação Em Saúde, Universidade Do Porto, Porto, Portugal; 3 INEB-Instituto de Engenharia Biomédica, Universidade Do Porto, Rua Alfredo Allen, 208, 4200-180 Porto, Portugal; 4 CAC ICBAS-CHP–Centro Académico Clínico Instituto de Ciências Biomédicas Abel Salazar–Centro Hospitalar Universitário de Santo António, Porto, Portugal; 5 Faculty of Health Sciences (FCS), Fernando Pessoa Hospital, University Fernando Pessoa (UFP), 4420-096 Porto, Portugal

**Keywords:** Physical activity, Exercise, Resistance training, Quality of life, Chronic lymphocytic leukemia, Oncology

## Abstract

**Background:**

This systematic review explores the impact of physical exercise on chronic lymphocytic leukemia (CLL) patients’ physical fitness, immunologic, and quality of life outcomes.

**Methods:**

Eligible studies were searched in PubMed and Web of Science up to February 2024 and were included if they involved participants in adult age, with confirmed CLL diagnose, using physical activity protocols with study design helding intervention protocols, clinical trials, or quantitative data reporting. Bias was assessed with ROBINS-I and RoB2 tools from Cochrane. The results are presented in tables and figures. A qualitative synthesis describes the main outcomes of the included studies. Meta-analysis was not performed due to significant heterogeneity.

**Results:**

This review identifies 92 studies, with 6 meeting the inclusion criteria, with a sample size of 323 patients. These studies focus on cardiovascular training combined with resistance training (four studies with total sample of 177 patients), continuous cardiovascular training (one study with 122 patients), and endurance resistance training (one study with 24 patients), highlighting the importance of physical exercise in CLL patients before treatment, with significant improvements in physical fitness and immunologic parameters on intervention groups.

**Discussion:**

The paucity regarding exercise influence on CLL, with small samples of patients in pilot study experiments, noted that exercise plays a vital role in improving physical fitness and immunologic parameters. However, none addressed strength training, which is known as one of the best options to increase muscular mass in physical activity interventions. The inconsistency of intervention and/or evaluation protocols ravels the advice of exercise and medical professionals on prescribing different modes of exercise, improving compliance with the prescribed exercise program, and determining which intervention in the context of exercise prescription should be used. More studies are needed to evaluate the impact of physical activity on the health-related quality of life and life span of the CLL patient.

**Trial registration:**

PROSPERO CRD42023464877.

## Introduction

Chronic lymphocytic leukemia (CLL) is a type of blood cancer described as a neoplasm composed of monomorphic small mature B cells that co-express CD5 and CD23 surface marker antigens [1], which leads to a progressive accumulation of monoclonal B lymphocytes [2]. In the diagnosis of CLL, CD5, CD19, CD20, CD23, surface or cytoplasmic kappa, and lambda light chains are regarded as essential markers, and CD10, CD43, CD79b, CD81, CD200, and ROR1 as additional targets useful in the differential diagnosis from other small B-cell lymphomas/leukemias [3].

CLL is the most common chronic leukemia among adults in Western countries, making up 25 to 35% of all leukemia cases in the USA [2, 4]. In Europe, leukemia has risen to become the eighth leading cause of cancer-related deaths since 2019, with an increasing tendency [2, 5]. With a male preponderance CLL represent 7% of non-Hodgkin lymphomas [1, 2], most frequently diagnosed between 65 and 74 years old, but individuals 45–64 already represent more than 30% of newly diagnosed cases [4]. Incidence rates are lower in the Asian and Black ethnic groups, compared with Caucasian due to genetic predisposition, environmental factors, healthcare disparities, or age distribution [5].

There are CLL-related mortality attributable risk factors that include elevated body mass index, occupational exposure to benzene, formaldehyde and to cigarette smoke [5]. However, when analysing data from low Social-Demographic Index areas, high body mass index showed a significant upward trend [5]. As the disease progresses, symptoms arise and include fatigue, shortness of breath during regular physical activity, lymph node enlargement, low-grade fever, unexplained weight loss, night sweats, feeling of fullness (related to enlarger spleen or liver) or infection of the skin, lungs, or sinuses [5, 6]. These symptoms impair physical fitness performance and daily activities, reducing the health-related quality of life. The diagnostic workup includes microscopic examination, immunophenotyping, fluorescence in situ hybridization (FISH), determination of CD38 and ZAP-70 expression, and the mutational status of IgHV genes in blood and/or bone marrow [6].

The most promising targeted therapies, including BCL-2 inhibitors and Bruton tyrosine kinase (BTK) inhibitors, are unable to offer a cure for CLL, while representing an economic burdensome with severe adverse events [5]. It is during the phase of active surveillance that physical activity may have an important role, putatively delaying symptoms and disease progression.

In chronic lymphocytic leukemia, prognostication has been supported by the Rai and Binet clinical staging systems, which rely on physical examination and standard laboratory tests [7, 8]. However, they lack genetic and molecular testing, giving rise to the CLL International Prognostic Index (CLL-IPI) which emerged as a more comprehensive prognostic framework [9–12] that combines genetic, biochemical, and clinical parameters into a five-categories prognostic model [13].

A less favourable prognostic outcome is associated with blood lymphocyte doubling time, complex karyotypes (Del 17q and Del 11q), expression of CD38, CD49d, and ZAP-70, and mutations on *TP53*, *NOTCH1*, and *SF3B1* genes [14, 15].

The survival of CLL cells strictly depends on a permissive and nurturing microenvironment [16, 17], which produces various proteins (chemokines, cytokines, and angiogenic factors) that interact with leukemic cells via surface receptors or adhesion molecules to support their survival and aggressiveness. On the other hand, the immune response against leukemic cells is compromised due to “exhaustion” of the cytotoxic T cells, and a suppressive immunological synapse formation with antigen-presenting cells [18]. Furthermore, natural killer (NK) cells acquire a phenotype that yields reduced ability to lyse CLL cells due to lack of cytoplasmic granules and impaired establishment of immunological synapse [16].

Intensive endurance exercise is known to induce lymphocyte apoptosis, which may affect immune function [19]. Regular resistance exercise decreases oxidative stress by enhancing antioxidant enzyme activity, which may lead to attenuated apoptosis-related proteins, such as caspase-3, BAX and BCL-2 expression [20]. Noteworthy, high-intensity interval training (HIIT) studies suggest that it is able to delete mainly differentiated T cells known to affect immunity to control latent infections, in contrast with continuous exercise that impacts survival of undifferentiated T cells, which might affect immunosurveillance and cytotoxic ability [21]. In recent years, scientific evidence revealed that skeletal muscle role in human physiology goes beyond the locomotor unit responsible for movement and posture, to include a major part on regulation in energetic and metabolic processes involved in the regulation of glucose and lipid metabolism, immunomodulation, anti-inflammatory, and inhibition of migratory mononuclear cells (such as macrophages and T-cells) towards the inflamed microenvironment (such as adipose tissue of obese persons) [22–24]. Other studies associated exercise-induced myokines production with the ability to increase immune-mediated cytotoxicity and the tumour infiltration by immune cells [25], growth and differentiation of muscle tissue promoting hypertrophy, providing energy substrate for contracting muscle fibres, activating mechanisms of energy production during fasting and improving tissue sensitivity to insulin, stimulating thermogenesis, glucose uptake by myocytes, and also contributing to an increase in bone mineral density [26]. Literature also demonstrated activation of NK cells after physical exercise [27, 28], with strenuous exercise increasing NK cytotoxicity during exercise, followed by a profound effect with 40% depression of the NK cell count even after cessation of exercise for as long as 7 days [29], with maximal exercise of large muscle groups leading to higher immune responses during repetitive bouts and elevated concentrations of leukocytes, neutrophilic granulocytes, and lymphocytes, resulting in leukocyte concentrations and the NK cell activity enhanced not only during exercise bouts, but also on the day after exercise [30]. A recent meta-analysis disclosed NK cell activity is largely elevated by acute physical exercise, with more significant effects in endurance versus resistance exercise and increasing with exercise intensity, providing solid evidence that elevated NK cell activity associated with exercise returns to baseline during the first 1–2 h of recovery, but not below the pre-exercise values, a proof that the functional change in NK cell activity exists independently from the quantitative change in NK cell count [31].

To address this complex landscape, many authors advocate for clarification of the effects of exercise on CLL, through the exploration of various exercise paradigms, including type, duration, and intensity, using state-of-the-art methods to detect immunological changes [32, 33]. In this review, we endeavour to review and comprehensively describe existing physical activity protocols for CLL patients and analyse their effects. Additionally, we aim to identify potential gaps in best practices related to the evaluation of physical activity, lifestyle patterns, and immunologic outcomes. Our findings will not only guide future research but also pave the way for innovative interventions in this crucial area.

## Methods

This systematic review was elaborated based on the methods outlined in the Cochrane handbook [34], and we also adhere to the Preferred Reporting Items for Systematic reviews and Meta-Analyses (PRISMA) guidelines [35] reporting standards. The purpose of this systematic review was to identify the relationship between physical activity (particularly HIIT and resistance training) and chronic lymphocytic leukemia, regarding fitness, health-related quality of life and feasibility, and immunologic parameters.

### Literature search strategy

This study was registered at PROSPERO database (CRD42023464877). Afterwards, automated searches were made in the PubMed and Web of Science databases up to February 2024, with no restriction on the initial date period. Free text terms were searched using the Boolean operators AND/OR applied to the title or abstract:

- **PubMed search key:** ((physical exercise) OR (aerobic training) OR (strength training) OR (exercise) OR (motor activity) OR (physical fitness) OR (physical activity) OR (intensive strength training) OR (resistance training) OR (plyometric exercise) OR (exhaustive exercise) OR (physical endurance) OR (exercise tolerance) OR (aerobic fitness) OR (strength endurance training) OR (strengthening exercise) OR (HIIT) OR (high intensity exercise) OR (high intensity interval training)) AND ((CLL) OR (Chronic lymphocytic leukemia) OR (leukemia)).
- **Web-of-Science search key:** TS = ((physical exercise) OR (aerobic training) OR (strength training) OR (exercise) OR (motor activity) OR (physical fitness) OR (physical activity) OR (intensive strength training) OR (resistance training) OR (plyometric exercise) OR (exhaustive exercise) OR (physical endurance) OR (exercise tolerance) OR (aerobic fitness) OR (strength endurance training) OR (strengthening exercise) OR (HIIT) OR (high intensity exercise) OR (high intensity interval training)) AND TS = ((CLL) OR (Chronic lymphocytic leukemia) OR (leukemia)).

In the case of Web-of-Science, we opted for field tag topic (TS) because it searches for topic terms in the title, abstract, and author keywords, making the search broader.

Articles were systematically screened (from September 2023 to February 2024), resulting in 92 hits after duplicates removal.

The eligibility criteria for this review included any study investigating exercise-based interventions in adult patients with chronic lymphocytic leukemia, following criteria according to population, intervention, comparison, outcomes and study design (PICOS) guidelines: Participants must be in adult age, with confirmed CLL diagnose; intervention must be with physical activity protocols; comparison between control group, or between different physical activity approaches; outcomes are the attributable risk in cancer or molecules numbers progression, overall quality of life and feasibility, or changes in morphologic, biologic, immunologic or physiologic characteristics; study design must held intervention protocols, clinical trials, or quantitative data reporting.

There were no restrictions regarding study design, with both clinical trials (randomized or non-randomized) and cohort studies being included. The focus was on studies evaluating the effects of exercise on clinical or functional outcomes in this population.

Articles must be written in English and available in full text. Gray literature was not included, but a systematic screening of selected papers bibliography was conducted to search for other articles that could be absent on our search. For this review we focused only on chronic lymphocytic leukemia. Variants, such as acute lymphocytic leukemia, myeloid leukemias, and lymphomas, were not considered due to differences in affected cells. No other inclusion or exclusion criteria were applied.

## Selection process

Two authors independently screened the retrieved records. Due to scarce literature retrieved, and the agreement in the manuscripts to include, there was no need for 3rd investigator for arbitrage. Automated removal of duplicates was performed using EndNote™ 20.3 for Mac (Clarivate™) but further manual removal was also used.

## Data extraction and methodological study quality assessment

The main characteristics of participants (age, gender, CLL staging described as Rai or CLL-IPI staging) and main study outcomes (quality of life, feasibility, exercise and biologic/physiologic outcomes variables), as well as their interaction, were identified and appraised.

Differences between baseline and post-protocol evaluation are described as mean ± standard deviation, in some cases mean (range), or as % of change compared to baseline (with mean ± SD also). On our tables, we opted to highlight the non-statistical differences between control and interventions groups, to enlighten the path for future investigations to fulfil the gaps of current knowledge.

The scientific quality of the included randomized clinical trials and observational studies was assessed independently by two reviewers using respectively CONSORT [36] and STROBE [37] checklist, as they are designed to ensure comprehensive reporting of observational research. Although CONSORT and STROBE are reporting guidelines, we used it as a proxy for assessing the completeness and transparency of reporting studies. In cases of discrepancies, a consensus-based final score was attributed after discussion. It was attributed a score of 0 (if do not meet the criterion) or 1 (if meets the criterion) for every item of the CONSORT and STROBE checklist. The global score was then converted into a percentage qualifying into 3 categories: A, study meets more than 80% of the criteria; B, study meets 50–80% of the criteria; and C, study meets less than 50% of the criteria [38].

Additionally, the risk of BIAS was assessed based on study type. For non-randomized cohort studies, the risk of bias in non-randomized studies of interventions (ROBINS-I) tool was applied [39], which evaluates bias across seven domains, including confounding, selection bias, and outcome measurement. For randomized trials, the Risk of Bias 2 (RoB 2) tool was used [40], which focuses on potential biases related to the randomization process, deviations from intended interventions, missing data, outcome measurement, and selection of reported results.

## Results

For this systematic review, a total of 1853 studies were identified within the search criteria. However, after screening titles and abstracts and applying inclusion/exclusion criteria (adult age patients, CLL malignancy, physical activity intervention protocols, after duplicates removal and excluding review articles) only 6 relevant articles were retrieved for full-text appraisal. Literature also shows associations of exercise in paediatric ages, but as the response to exercise is different in adults, those studies were disregarded while screening titles. One other study [41] was not included because it addressed a study-case with an unstructured exercise intervention protocol, without the assessment of initial and end physical fitness outcomes. Among the selected studies, 3 studies were randomised clinical trials (RCT’s) with physical activity (PA) intervention and without any prior treatment to CLL disease; 2 studies were pilot studies with CLL patients, and 1 interventional study with physical activity intervention but with previous or ongoing CLL treatment.

**Fig. 1:**
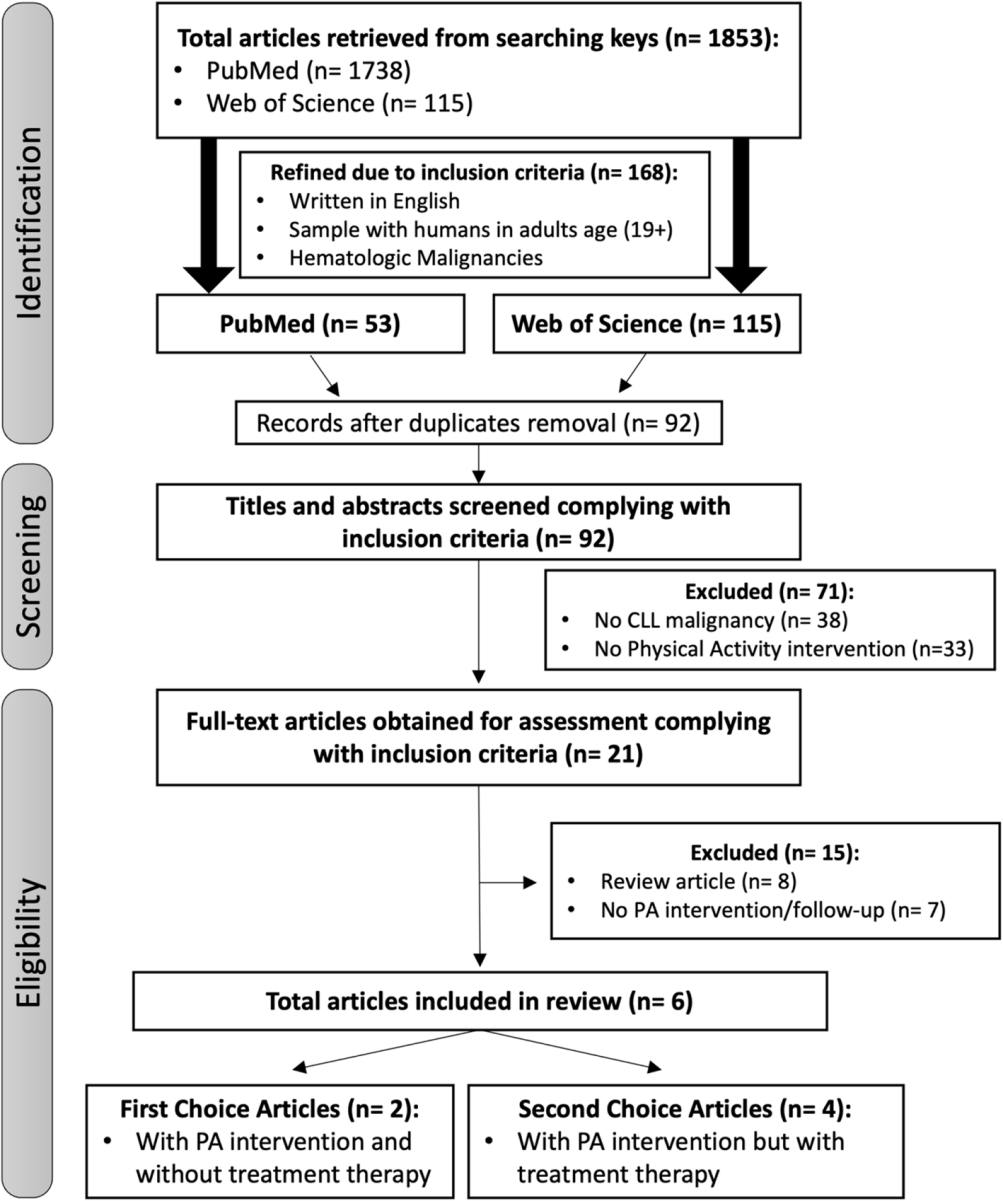
PRISMA flow diagram

In the studies included, physical activity with different types and intensities (aerobic, strength, resistance and HIIT) were objectively assessed. Because CLL exercise intervention remains an unclear field of expertise, literature addressing PA influence in CLL patients (exclusively) disease parameters is rare (only one interventional study and two small Pilot Studies with 24 patients [32], 16 patients [33], and 15 patients [42]), reason that led us to select for inclusion any exercise intervention study that included CLL adult patients in the sample [43–45]. Figure 1 schematize the search flow diagram.

Table 1 summarizes the quality assessment of each study, according to CONSORT [36] and STROBE [37] guidelines (as they are designed to ensure comprehensive reporting of observational research, they were used as a proxy for assessing the completeness and transparency of reporting studies); the risk of BIAS was accessed with ROBINS-I tool for non-randomized interventions [39], and the RoB 2 tool for randomized controlled trials [40]. Although study quality of the selected studies were graded A or B, the authors did not include an estimation of BIAS direction and magnitude, for example, regarding differences in the medical surveillance of participants, physical activity surveillance, follow up of daily activities, and only focused on details about additional data they collected to tackle this problem. The most impactful domains in non-randomized studies for overall risk of BIAS were BIAS due to confounding, while in randomized studies were BIAS due to randomization process and in measurement of outcomes. One study from Crane et al. [32] revealed not only Serious risk of BIAS on controlling confounding, but also in classification of intervention, deviation from intended interventions, missing data, and selection of reported result. Knowing the limitations of a home-based approach, it is not clear how the research team controlled the implementation of exercise protocol between groups, and even the BIAS of the selected outcomes (6 min walk test, timed up-and-go, chair stand test, and FACT questionnaires) could be critically contaminated upon measuring; and without a control group to compare to. For primary outcome VO2 assessment, most of the studies have used standardized protocols or gold standard physical evaluation equipment’s to reduce BIAS, but regarding muscular evaluation, only Person et al. [45] have attempted that BIAS reduction. In all remaining studies were used evaluation tests subjected to major risk of BIAS, mainly due to missing measurement of outcome BIAS control. By using non gold standard physical evaluation while applying questionnaires (inherently subjective) to evaluate secondary outcomes like leisure time activity, physical and functional well-being, the risk of BIAS is greatly augmented.

**Table 1:** Study quality according to CONSORT [36] and STROBE [37] guidelines, and risk of bias according to ROBINS-I tool [39] for non-randomized interventions and RoB 2 [40] for randomized clinical trials. HL Hodgkin’s *l*ymphoma*,* NHL *non-*Hodgkin’s *l*ymphoma

		**STROBE**	**ROBINS-I**							
**Study**	**Pathology**	**Study quality**	**BIAS due to confounding**	**BIAS in selection of participants**	**BIAS in classification of interventions**	**BIAS due to deviations from intended interventions**	**BIAS due to missing data**	**BIAS in measurement of outcomes**	**BIAS in selection of reported result**	**Overall risk BIAS**
**Crane et al. (2023)**	CLL	B	Serious	Low	Moderate	Serious	Moderate	Low	Serious	Serious
**Artese et al. (2022)**	CLL	B	Moderate	Low	Low	Low	Low	Low	Low	Moderate
**Macdonald et al. (2021)**	CLL	A	Low	Low	Low	Low	Low	Low	Low	Low
		**CONSORT**	**RoB 2**							
		**Study quality**	**BIAS due to randomization process**	**BIAS due to deviations from intended interventions**	**BIAS due to missing outcome data**	**BIAS in measurement of outcomes**	**BIAS in selection of reported result**	**Overall risk BIAS**		
**Persoon et al. (2017)**	Multiple myeloma and lymphoma	A	Low	Low	Low	Some concerns	Low	Some concerns		
**Furzer et al. (2016)**	HL, NHL and myeloma	B	Some concerns	Low	Low	Low	Low	Some concerns		
**Courneya et al. (2009)**	HL and NHL	A	Low	Low	Low	Low	Low	Low		

From the 326 participants included in the selected studies, only 22.1% of the sample corresponds to CLL patients, 54.6% refers to non-Hodgkin’s lymphoma, 19.0% refers to multiple myeloma, and 4.3% refers to Hodgkin’s lymphoma. Most of the studies include patients with median age around 50 years old, with body fat above the limits for age [46], and BMI in overweight range. Time for inclusion after diagnosis ranged between 2.1 ± 2.6 and 7.9 ± 9.1 years. In the two studies that engaged CLL patients without previous treatment [33, 42], patients recruited were in Rai stage 0 or I (83.3% of patients in intervention group and 80.0% in control group, remaining percentage are described as unknown) or CLL-IPI stage 0–1 and 2–3 (50.0% of patients in intervention group and 30.0% in control group, remaining percentage are described as unknown). In the remaining studies, this information is not addressed by the authors. Table 2 summarizes the baseline overview of the population.

**Table 2:** Baseline overview of the population. Data are presented as mean ± standard deviation or as median (range). HIIT high-intensity interval training. BMI body mass index

	**Crane et al. (2023)**		**Artese et al. (2022)**		**Macdonald et al. (2021)**		**Persoon et al. (2017)**		**Furzer et al. (2016)**		**Courneya et al. (2009)**	
	Intervention protocol	Baseline	Intervention protocol	Control	Intervention protocol	Control	Intervention protocol	Control	Intervention protocol	Control	Intervention protocol	Control
**Intervention characteristics**	Home-based aerobic only or aerobic with resistance exercises		HIIT	Usual care	HIIT	Usual care	HIIT + resistance Ex	Usual care	Aerobic + resistance Ex	Usual care	Aerobic exercise training	Usual care
**Demographics**												
Age (yrs.)	63.04 ± 8.67		62.2 ± 9.4	66.5 ± 7.1	63.9 ± 10.8	66.5 ± 7.1	53.5 (20–67)	56 (19–67)	48.2 ± 12.3	49.6 ± 14.1	51.2 (18–77)	53.5 (18–80)
Sex (m/f)	16/8		3/6	4/2	4/6	4/2	32/22	37/18	18 (unknown m/f ratio)	19 (unknown m/f ratio)	37/23	35/27
BMI (kg/m^2^)	27.4 ± 5.07	27.8 ± 4.83	27.3 ± 7	25.4 ± 3.1	27.3 ± 6.6	25.4 ± 3.1	25.6 ± 4.6	25.1 ± 3.6	24.2 ± 3.3	25.8 ± 4.7	27.4 ± 4.5	26.7 ± 5.4
Lean mass (kg)					48.2 ± 12.4	53.7 ± 10.5			48.34 ± 10.33	46.85 ± 10.21	52.4 ± 10.9	49.9 ± 10.0
Body fat (%)					37.7 ± 10.3	31.0 ± 10.3			29.79 ± 9.20	34.56 ± 11.16	32.6 ± 9.9	32.6 ± 10.9
**Disease characteristics**												
Chronic lymphocytic leukemia (N/total sample)	24/24		9/9	6/6	10/10	6/6	Unknown/54	Unknown/55	Unknown/18	Unknown/19	4/60	10/62
Time since diagnose (yrs)			7.9 ± 9.1	4.6 ± 4.3	7.3 ± 8.7	4.6 ± 4.3					2.1 ± 2.6	2.8 ± 3.3
Prior treatment (N/total sample)	9/24											
Ongoing treatment (N/total sample)	12/24											
Rai stage (N)												
0	7		6	4	7	4					16	18
I +	17		1	1	1	1					43	43
Unknown			2	1	2	1					1	1
CLL-IPI (N)												
0–1					2	2						
2–3					1	1						
Unknown					7	3						

The duration of intervention was predominantly 12 weeks with 2–3 training days per week, except for the Persoon et al. [45] intervention which lasted 18 weeks, and Crane et al. [32] that lasted 16 weeks. The main outcomes were associated with health-related quality of life questionnaires (reported in 4 studies), objective measured physical activity (reported in 4 studies), and immune characteristics (reported in 2 studies). On physical activity outcomes, the most analysed parameter was the cardiovascular outcomes, but with different characterizations. Crane et al. [32] used a home-based exercise program using 16 combinations of exercises but do not clearly describe the frequency, intensity, time and type (FITT) principles of exercise prescription. Artese et al. [42] did not present data about this parameter but concatenated to another study [33] where data was collected from. Equipment’s used were treadmill and upright or recumbent cycle ergometer. Regarding muscular parameters, Courneya et al. [43] did not present any outcome, while others presented data regarding muscle strength, muscle endurance, or maximal torque. It was also assessed the physical activity with accelerometry technology in Persoon et al. [45], and leisure time activity (by questionnaire) as well as bone mineral density in the study by Furzer et al. [44]. Table 3 presented the characteristics of the included studies with physical intervention.

**Table 3:** Characteristics of the included studies with intervention. ND not described, MSEC maximal short exercise capacity (using steep ramp test), 1RM 1 repetition maximum, HRR heart rate reserve, CV cardiovascular training, HIIT high-intensity interval training, REx resistance exercise

Study	Sample size	Exercise duration and frequency	Aerobic mode	Aerobic intensity	Aerobic interval	Aerobic recovery	Resistance mode	Resistance intensity	Resistance interval	Resistance recovery	Supervised OR unsupervised
Crane et al. (2023)	24	16 weeks, combination of home-exercise routines	ND	ND	ND	ND	ND	ND	ND	ND	Unsupervised and self-reported
Artese et al. (2022)	15	12 weeks, 3 × /week (2 × HIIT + REx and 1 × only HIIT)	Treadmill	Warm-up: 5 min Work phase: 80–90% HRR Cool down: 5 min	60–90 s work followed by 60–90 s active recovery until complete máx 20 min	50–60% HRR	Major muscle groups using machines (leg press, chest press, seated row)	Warm-up: 10 rep at 40–60% of 1RM Work phase: Many reps possible at 70% of 1RM for 2 sets	ND	ND	Supervised by exercise physiologist
Macdonald et al. (2021)	16	12 weeks, 3 × /week (2 × HIIT + Rex and 1 × only HIIT)	Treadmill	Warm-up: 5 min Work phase: 80–90% VO2 reserve Cool down: 5 min	60–90 s work followed by 60–90 s active recovery until complete máx 20 min	50–60% VO2 reserve	Major muscle groups using machines (leg press, chest press, seated row)	Warm-up: 10 rep at 40–60% of 1RM Work phase: Many reps possible at 70% of 1RM for 2 sets	ND	ND	Supervised by exercise physiologist
Persoon et al. (2017)	109	18 weeks, from 1–12 weeks 2 × /week From 13–18 weeks 1 × /week	Cycle ergometer	65% MSEC	2 × blocks of 8 min (MSEC) Week 1–8: 30 s work 60 s active rest Week 9–18: 30 s work 30 s active rest	30% MSEC	4 standardized exercises (vertical row, leg press, bench/chest press, pullover/flies) and 2 exercises for abdominal muscles and upper legs	Week 1–12: 65–80% 1RM Week 13–18: 35–40% 1RM	Week 1–12: 2 sets of 10 rep Week: 13–18: 2 sets of 20 rep	ND	Supervised by instructed physiotherapists
Furzer et al. (2016)	37	12 weeks, 3 × /week	ND	Initial phase: 50% of HRmax Progression phase: 5% weekly increment until reach 85% HRmax	Max 30 min continuous CV training		6 machines and 2 dumbbell targeting major muscle groups	Initial load: 50% 1RM Aimed load: 80% 1RM	Initial load: 3 sets of 10–15 rep Aimed load: 2 to 3 sets of 6–8 rep	Minimum of 90 s recovery between sets	Supervised by exercise physiologist
Courneya et al. (2009)	122	12 weeks, 3 × /week	Upright or recumbent cycle ergometer	60–75% VO2 peak	15–45 min continuous CV training						Supervised by exercise physiologist

Although meta-analysis was not feasible due to the heterogeneity of interventions, populations, and outcome measures across the included studies, the following tables summarize key findings and trends observed in the data. The results are presented focusing on physical parameters and health-related quality of life (HRQoL) outcomes, with particular attention to the effects of the interventions.

Regarding physical parameters, the included studies report a variety of physical outcomes, including VO2peak, muscle strength, body composition, and functional tests (e.g., 6-min walk test, timed up and go). Across studies, most results show improvements in the intervention groups compared to control, though not all reach statistical significance. Macdonald et al. [33] and Persoon et al. [45] observed significant improvements in VO2 peak in the intervention groups compared to controls (from 21 ± 04 to 22 ± 07 L/min and from 24 ± 7 to 28 ± 7 mL/kg/min, respectively), indicating enhanced aerobic capacity after HIIT and resistance training. Several studies noted increases in muscle strength, particularly in leg and chest press exercises. Even Crane et al. [32] home-based exercise intervention reported an increase from 112 ± 37 to 153 ± 46 kg in leg strength. These improvements suggest positive adaptations to both aerobic and resistance exercise protocols. Regarding body composition, there were no significant changes in body fat percentage or lean mass in most studies. This indicates that while functional and aerobic capacity improved, body composition remained relatively stable across interventions. Table 4 summarizes the objectively assessed physical parameters for each study.

**Table 4:** Objectively assessed physical parameters described as mean ± standard deviation. Bold text indicates non-statistical differences between groups. Because of the risk of recruitment bias, MacDonald et al. (2021) study did not report *p*-values throughout, so bold text represents small-medium effects size < 0,5 Hedges’G

	**Crane et al. (2023)**		**Macdonald et al. (2021) (and Artese et al. (2022))**		**Persoon et al. (2017)**		**Furzer et al. (2016)**		**Courneya et al. (2009)**	
	T0	T1	T0	T1	T0	T1	T0	T1	T0	T1
**Leisure time activity (questionnaires)**							816 ± 537	1964 ± 1236		
**Accelerometry (cpm)**					**189.0 ± 79.2**	**241.4 ± 92.3**				
**PASE-score**					**96.9 ± 99.5**	**131.3 ± 82.4**				
**VO2 peak (ml/kg/min)**			**26.7 ± 6.1**	**28.5 ± 5.5**	**21.7 ± 4.8**	**26.0 ± 6.3**			24.7 ± 7.2	29.4 ± 8.6
**VO2 peak (L/min)**			**2.1 ± 0.4**	**2.2 ± 0.7**					2.02 ± 0.66	2.38 ± 0.81
**W peak (Watt/kg)**					**2.0 ± 0.5**	**2.4 ± 0.7**	1.45 ± 0.44	1.84 ± 0.48		
**6 min walk test (m)**	**500.15**	**525.08**								
**Timed up and go (s)**	**5.58**	**5.67**								
**Muscle strength**										
Leg			**133.5 ± 30**	**137.9 ± 53.5**			112 ± 37	153 ± 46		
Chest			**48.8 ± 20.2**	**43.2 ± 26.9**			32 ± 18	47 ± 25		
Back			65.7 ± 27.8	51.2 ± 24.9			52 ± 18	67 ± 24		
Arms							9 ± 4	12 ± 6		
**Muscle endurance (max rep with 70% 1RM)**										
Leg			**20.6 ± 4.9**	**20.3 ± 5.7**						
Chest			**14.8 ± 1.7**	**14.4 ± 3.5**						
Back			**15.8 ± 1.3**	**15.7 ± 3.8**						
**Chair stand test (number rep)**	**13.5**	**13.5**			**15.5 ± 4.6**	**18.7 ± 6.0**				
**Grip strength (kg)**	**9.67**	**14.00**			**35.5 ± 10.7**	**40.9 ± 12**				
**Maximal torque m. quadríceps (Nm)**					**145.6 ± 51.4**	**173.9 ± 55.9**				
**Bone mineral density**										
AP spine (after 24 wk)							1.226 ± 0.185	1.232 ± 0.180		
Femur (after 24 wk)							1.061 ± 0.170	1.063 ± 0.170		
**Lean mass (kg)**			**48.2 ± 12.4**	**3.1 ± 5.2 (% change)**			48.34 ± 10.33	50.07 ± 11.35	52.4 ± 10.9	52.3 ± 11.5
**Fat mass (kg)**									**25.8 ± 9.5**	**26.0 ± 10.1**
**Body fat (%)**			**37.7 ± 10.3**	** − 4.9 ± 12.5 (% change)**	SUM SKF ⇑	SUM SKF ⇑	29.79 ± 9.20	28.68 ± 10.56	**32.6 ± 9.9**	**32.8 ± 10.1**

Regarding health-related quality of life (HRQoL), a wide range of outcomes was reported using multiple instruments [47–52] such as the EORTC QLQ-C30 [53], symptom assessment scale [54], and functional assessment of cancer therapy [55]. Across studies, improvements were noted, particularly in physical functioning, role functioning, and fatigue, though again, not all differences were statistically significant. Crane et al. [32] reported significant improvements in fatigue scale (from 37 (IQR, 27.75; 45.75) to 45 (IQR, 39.50; 47.75)). These findings highlight the potential of structured exercise interventions to enhance both physical functioning and overall QoL in cancer survivors. Furzer et al. [44] noted positive trends in the FACT-Lym total score (from 81.2 ± 12.8 to 90.2 ± 10.1), suggesting improvements in both physical and emotional well-being following aerobic and resistance exercise training. Courneya et al. [43] also found significant statistical differences in FACT-An total score, and in happiness scale and depression scale, reflecting the importance of physical activity in mental health and HRQoL well-being. Table 5 summarizes the HRQoL outcomes.

**Table 5:** Health-related quality of life outcomes. Data are shown as sum ± std dev or as median (interquartile range). *IQR* interquartile range. Bold text indicates non-statistical differences between groups

	**Crane et al. (2023)**		**Artese et al. (2022)**		**Macdonald et al. (2021)**		**Persoon et al. (2017)**		**Furzer et al. (2016)**		**Courneya et al. (2009)**	
	T0	T1	T0	T1	T0	T1	T0	T1	T0	T1	T0	T1
**HADS–anxiety **[47]							**4.4 ± 3.5**	**4.1 ± 4.1**	**5.3 ± 2.9**	**5.0 ± 3.9**		
**HADS–depression **[47]							**3.8 ± 3.8**	**3.2 ± 3.4**	**4.7 ± 3.4**	**2.4 ± 2.2**		
**SCFS–fatigue **[48]									13.7 ± 4.4	9.3 ± 2.0		
**PCS (SF-36)-physical component **[49]									43 ± 10	52.6 ± 6		
**Anxiety **[50]											**18.4 ± 6.6**	**16.5 ± 5.2**
**Depression **[51]											7.7 ± 5.7	5.4 ± 4.5
**Happiness scale **[52]											59 ± 22.4	70.9 ± 16.4
**EORTC-QLQ30 **[53]												
Global quality of life							**62.2 ± 18**	**75.0 ± 18.7**				
Physical functioning							**74.5 ± 18.3**	**83.1 ± 19.1**				
Role functioning							**60 ± 31.6**	**81.0 ± 23.8**				
Emotional functioning							**83.2 ± 14.9**	**86.2 ± 16.3**				
Cognitive functioning							**87.3 ± 15.6**	**83.7 ± 18.0**				
Social functioning							**71.7 ± 27.2**	**86.0 ± 20.3**				
Disease symptoms							**16.6 ± 13.1**	**18.7 ± 12.8**				
**Fatigue (MFI) **[54]												
Physical fatigue							**13.2 ± 4.2**	**9.8 ± 4.4**				
General fatigue							**12.7 ± 3.8**	**10.0 ± 4.5**				
Mental fatigue							**10.0 ± 4.3**	**9.7 ± 4.5**				
Reduced activity							**12.2 ± 4.1**	**9.6 ± 3.9**				
Reduced motivation							**8.8 ± 3.8**	**8.0 ± 3.2**				
**FACT **[55]												
Physical well-being			**24.9 ± 3.2**	**25.6 ± 2.1**								
Social well-being			**23.0 ± 3.2**	**24.6 ± 2.9**								
Emotional well-being			**19.6 ± 2.7**	**20.6 ± 2.2**								
Functional well-being			21.7 ± 3.4	23.9 ± 3.2								
Lymphoma subscale			**49.3 ± 3.9**	**53.3 ± 4.2**							**47.4 ± 8.7**	**50.3 ± 7.2**
FACT-G total			**89.2 ± 6.6**	**94.6 ± 5.5**					81.2 ± 12.8	90.2 ± 10.1		
FACT-lym total			**138.6 ± 9.4**	**147.9 ± 6.7**								
FACT-An total											140.7 ± 26.8	151.4 ± 21.7
FACT fatigue	Median 37.00 IQR (27.75; 45.75)	Median 45.00 IQR (39.50; 47.75)										

While statistical significance was not reached in all studies, particularly due to small sample sizes, the effect sizes (Hedges’ G) suggest that moderate improvements were achieved in most physical and QoL outcomes. The results in bold within the tables indicate non-significant findings, yet effect sizes suggest potential clinical relevance, especially in aerobic capacity and muscle strength.

Although meta-analysis was not performed, the overall trends observed in these studies support the effectiveness of combined aerobic and resistance exercise interventions in improving aerobic capacity, muscle strength, and quality of life in cancer patients and survivors. However, the small sample sizes and the variability in outcomes measured across studies limited the ability to conduct a formal meta-analysis. The results nonetheless indicate positive trends that warrant further investigation in larger, more homogeneous samples.

## Discussion

This systematic review sought to critically assess the available evidence on the relationship between physical activity (particularly HIIT and resistance training) and chronic lymphocytic leukemia, regarding fitness, health-related quality of life and feasibility, and immunologic parameters. Only 6 papers met our search criterion, with 2 addressing the HIIT type of physical activity intervention. Meta-analysis was not feasible due to the heterogeneity of interventions, populations, and outcome measures across the included studies but key findings and trends are reported.

Most of CLL studies were conducted throughout the last two decades, and before 2009, no intervention studies were reported, only observational using questionnaires and involving anthropometric parameters such as height, weight, waist and hip circumference, or the body mass index [56–58]. And because we are still in an early bird phase, many methodological problems are biasing results. Early meta-analysis in 2009 addressed resistance training in cancer survivors and displayed post training improvements in cardiopulmonary function from 6 to 39%, and increases in muscle strength between 11 and 110% [59]. We hypothesize that this disparity may be explained by factors such as the contribution of learning effect, variability in strength exercises, intensities and duration of exercise programs, genetic differences, type and stage of cancer, diverse cancer treatments, and the time elapsed since cancer diagnosis. This shows the importance of knowing the progress path between sedentary behaviour and physical active behaviour, and the interconnection with patient’s physical active before and after diagnosis.

There is general agreement in the absence of adverse training effects on immunological, endocrinological, and hematopoietic variables, or lymphedema. Moreover, it seems that even high training intensities have been well-tolerated in cancer survivors [59].

A recent systematic review and meta-analysis confirmed that, in addition to clinical stage evaluation [60], the best practice to initial prognosis in CLL patients should include the chronic lymphocytic leukemia international prognostic index (CLL-IPI), which combines 5 parameters (age, clinical stage, *TP53* status, IGHV mutational status, and serum B2-microglobuli) [61]. In the studies of our review, only Macdonald et al. (2021) implemented CLL-IPI, while Artese et al. (2022) and Courneya et al. (2009) used Rai staging.

The consistency of “best practice on evaluation” in the physical fitness evaluation is not met, leading to inconclusive data and poor strength evidence. Investigations until this date support intensive endurance exercise, known to induce lymphocyte apoptosis affecting the immune system [19]; regular resistance exercise, who can decrease oxidative stress which may lead to attenuate apoptosis related protein [20]; high-intensity interval training suggesting that it may be able to delete mainly highly differentiated T cells, known to affect immunity to control latent infections [21]. However, for the same physical fitness outcomes, cardiovascular protocols differ in equipment (treadmill or recumbent cycle ergometer); in intensity (90% VO2 reserve, or 90% HR reserve, or 65% maximal short exercise capacity, or HR maximal, or VO2 peak); on duration (20 min interval training, 15–45 min continuous, 30 min continuous, 8 min intervals); and on recovery (60–90 s active recovery, 30–60 s active recovery, no recovery). Muscular protocols differ in resistance mode (major muscle groups using machines, or dumbbells, or calisthenic exercises); resistance intensity (70% of 1-RM for maximal repetitions, 80% of 1-RM for 6–8 repetitions, 65–80% 1-RM); duration of effort (2 sets of 10 repetitions, 2–3 sets of 6–8 repetitions, 2 sets of 20 repetitions); and recovery (minimum of 90 s between sets, or not described at all). For the goodness of proof, it is mandatory that gold-standard equipment’s are used, such as isokinetic dynamometers to evaluate muscular effort, cardiopulmonary exercise test (CPET) with electrocardiogram to assess cardiac and pulmonary response, dual-energy X-ray absorptiometry (DEXA) to evaluate corporal composition.

In the studies with intervention protocols available, one was based only on recumbent cycle ergometer training, regarding heart rate reserve as modulator of intensity [43], three studies with HIIT exclusively during 30 min, or with 1 h combining 30 min HIIT plus 30 min resistance training [33, 42, 45], and another study using continuous cardiovascular training concomitant with resistance training [44]. However, because there is a combination of continuous cardiovascular (or HIIT) plus RT, we cannot fully establish a relation between resistance/strength training only, and cardiovascular only, or the combined effect of training in CLL patients, neither infer about the difference between endurance and strength training in resistance training protocols. Nevertheless, findings showed that more than 12-weeks of training, with more than 30 min. of HIIT combined with muscle endurance-based resistance training for treatment naïve CLL patients, is feasible and is associated with significant effects on muscle strength and normal immune cell functions [33, 42, 45].

Corroborated from current knowledge, urges the need to clarify the exercise characteristics and outcomes regarding HIIT, resistance training (also between strength and endurance approaches) and combined training in CLL patients. The difference between muscular endurance training (more repetitions, and fatigue resistance to exercise) and maximum strength training (increment in muscle strength and/or muscle hypertrophy) must also be assessed. Most studies use DEXA and BIA for body composition analysis, which are indirect measures, insensitive to changes in muscle size, that consequently would keep very small changes from muscle hypertrophy adaptations undetected [62]. As described elsewhere [63, 64], when volume is equated, resistance training frequency does not significantly or meaningfully impact muscle hypertrophy. However, higher training frequencies can help to accumulate more significant volumes of training, which may enhance hypertrophic response.

The studies obtained in this review shows a preference for low-moderate intensities, or endurance effort on resistance training (65–70% of 1-RM), but as literature suggest, exercise-induced myokines production have a critical role in increasing cytotoxicity and the infiltration of immune cells into the tumour [25]. When comparing aerobic only, strength only and both guidelines, the hazard ratio for all-cause mortality was lower when combining both guidelines, but when alone, strength training was better than aerobic; in the case of cardiovascular disease mortality, both guidelines combined are still the better choice to a lower hazard ratio; but in cancer mortality, strength only training shows to be the better option compared to combined guidelines [65]. There are no studies addressing the use of strength training on CLL patients but given the fact that one of the most evident symptoms as the disease progresses are fatigue and shortness of breath during regular physical activity, it seems reasonable that a strength training is better tolerated than a cardiovascular training. Confirming the feasibility and success of this type/mode of training should be engaged in future studies.

It is also important to objectively assess the daily lifestyle of patients to establish a possible etiology of CLL, implementing the accelerometry technology, to ensure correct lifestyle patterns recommendations, and make a connection point with anatomic/morphologic data (muscular mass, body fat percentage, weight, blood, and cardiovascular parameters), and physical condition (VO2, strength, flexibility, mobility, and more).

Despite the medical conservative approach in leukemic patients regarding exercise prescription, leading exercise oncologists suggest that all cancer patients should avoid inactivity and engage in safe exercise training [66]. It was reported in a small cohort with 12 patients with hematologic disease (7 patients with acute myeloid leukemia, 1 acute lymphoblastic leukemia and 4 non-Hodgkin’s lymphoma) receiving high-dose chemotherapy, that none of the patients with thrombocyte counts below 10.000/μl suffered bleeding and no patient with haemoglobin counts below 8 g/dl suffered critical tachycardias, showing that physical exercise in patients with severe cytopenia is safe and results in increased physical performance and unchanged quality of life [67]. Also in the studies we collected, the feasibility of the intervention protocols were always highly rated, with 99 ± 3.6% attendance and 148.5 ± 5.4 min/week of exercise and with 100% safety [33, 42, 45]; with 91% attendance with 88 ± 17 min/week (Furzer et al., 2016); with small adverse events related with intervention protocol such as back, hip and knee pain (Courneya et al., 2009) but without impairment of patients maintenance on the protocol.

Considering immunologic outcomes, only two small pilot studies compared disease parameters. One revealed a significant effect on NK-cells, including increased absolute counts, cytotoxic function, as well as increased perforin and granzyme B expression, mimicking the response to exercise in subjects without cancer [33] while the other enlightened an increased ratio CD4:CD8 T-cell, and reduced proportion of T-cells subsets [32]. Although these results should be treated with care since a control group is missing, and patients received treatment before or during intervention with medication known to alter T-cell phenotype and function, this relationship between exercise and immune outcomes should be further explored in future studies. As reported elsewhere [68], exercise is an effective modulator in sustaining proliferative signalling, evading growth suppressors, resisting cell death, enabling replicative immortality, inducing angiogenesis, activating invasion and metastasis, exercise and tumour-promoting inflammation and reprogramming energy metabolism, evading immune destruction, with myokines playing a significant role in the prevention of cancer proliferation. The effect of exercise in these hallmarks of cancer should be evaluated in CLL cells.

## Conclusion

This systematic review exposed the gap of few implemented studies and conflicting results in some physical activity approaches in CLL patients. To our knowledge, only three studies specifically addressed CLL patients, but with small samples of patients in pilot study experiments. Nevertheless, it is noted the vital role of exercise before treatment, with significant improvements in physical fitness, and immunologic parameters.

Aerobic exercise training significantly improved objective physical function when worked at 60–75% of VO2 peak; aerobic and resistance exercises combined resulted in significant clinical improvements in cardiorespiratory fitness when worked at 85% HRmax and 80% of 1-RM; HIIT combined with muscle endurance-based resistance training is feasible and associated with large effects on muscle strength and immune function when worked at 90% VO2 reserve and 70% of 1-RM; even a home-based physical activity program increased leisure-time PA and decreased fatigue. However, no significant beneficial effects of supervised high-intensity exercise programs on physical fitness and fatigue with intensities of 65% HR and 40% of 1-RM. This suggests that this population do not differ from healthy populations, and it seems to be more beneficial to adjust the intensity of workout to higher limits rather than low limits. Studies addressing strength-based resistance training are inexistent, since all the studies analysed just used endurance-based resistance training programs, or combined programs.

The present literature also shows a gap regarding evaluation process, advising that future studies should include most accurate instruments to address disease parameters: physical exam and health history, complete blood count, blood chemistry studies, lactate dehydrogenase testing, beta-2-microglobulin testing, flow cytometry to quantify tumour markers at cell surface, FISH analyses, gene mutation testing, serum immunoglobulin testing, chest x-ray for organs and bones, CT scan and/or PET-CT scan. Additionally, include reference instruments to evaluate physical fitness components objectively: DEXA scan for body composition, dynamometers for muscular strength or endurance, VO2max protocol with ergospirometry and ECG, O2 saturation, lactate, ultrasound for muscle density, blood pressure, heart rate and accelerometry. The physical activity must be precisely quantified to define the treatment dose of exercise.

The conclusions mentioned here are supported by a short number of studies. Therefore, care should be taken to interpret these findings. To be able to advise Exercise Professionals to prescribe different modes/types of exercise, improving compliance with the prescribed exercise program and determine which intervention in the context of exercise prescription should be used to maximize the benefits resulting from the regular practice of physical activity, more studies are needed to evaluate the impact of PA in the health-related quality of life and life span of the CLL patient.

## Data Availability

All data is available online and described in references.
